# Rats as pets: Predictors of adoption and surrender of pet rats (*Rattus norvegicus domestica)* in British Columbia, Canada

**DOI:** 10.1371/journal.pone.0264262

**Published:** 2022-02-18

**Authors:** Cheng Yu Hou, Alexandra Protopopova

**Affiliations:** Animal Welfare Program, Faculty of Land and Food Systems, The University of British Columbia, Vancouver, British Columbia, Canada; University of Lincoln, UNITED KINGDOM

## Abstract

Whereas much research has been conducted on rats in their roles as pests and laboratory animal models, little is known about rats in their role as companion animals. However, rats have become the third most common companion animal admitted to the British Columbia Society for the Prevention of Cruelty to Animals (BC SPCA) shelter system after cats and dogs. This paper analyses 5 years of province-wide rat admission and outcome data (n = 3,392) at the BC SPCA. Most rats that entered BC SPCA shelters were white, sexually intact, and pups less than 6 months old. Rats were mostly relinquished by their owners, and the most common surrender reasons were due to owner-related issues and housing issues. Reasons for euthanasia were primarily poor health and neonatal age. A multiple linear regression model found that rats that were either senior, albino, unhealthy, seized by humane officers, or born onsite tended to stay longer in shelters (F[12, 1466] = 9.565, p < .001, adjusted R^2^ = .06). Time to adoption for albino rats was 79% longer than for white rats. These findings help us understand the preferences of rat adopters and why the rat-human relationship may fail. Results may also be useful to improve the quality of life for pet rats by identifying programs to reduce their length of stay in animal shelters. Finally, our study highlights new questions for welfare research in an understudied companion animal–the pet rat.

## Introduction

Human perception of the value of animal life is dependent not only the species but also the context of the human-animal interaction [1]. This contradictory human-animal relationship is particularly interesting in the brown rat (*Rattus norvegicus*) due to their prolonged co-existence with humans. Whereas rats are widespread throughout human civilization, they carry a bad reputation as a reservoir of zoonotic pathogens and a competitor for human food [2]. At the same time, the domesticated fancy rat (*Rattus norvegicus domestica*) has a long history as a companion animal [3], but their popularity as a pet is relatively low compared to more traditional pets such as cats and dogs [4, 5]. In Australia, approximately 2.7% of households kept small mammals as pets, and 19% of them were rats and mice [5]. The proportion of pet rodents was much smaller than dogs (39.9%), cats (27%), and birds (9%). From the evolutionary perspective, it is logical that people have disdain and avoid animals that are harmful or carry pathogens that are potentially harmful [6]. Moreover, traditional beliefs that describe rats as a mean, cunning, and devious character strengthen the negative attitudes toward keeping them as companion animals [6]. Consequently, not much research has been conducted on pet rats since they are not as popular as other companion animals.

Nevertheless, over the past 5 years, the population of pet rats at the British Columbia Society for the Prevention of Cruelty to Animals (BC SPCA) was, while much smaller than companion animals like cats and dogs [4], is now the third most common animal surrendered [7]. The lack of understanding about pet rat husbandry and species-specific needs could result in poorer care of these animals in pet homes and animal shelters. Furthermore, Ceríaco [8] concluded that negative values and folklore about amphibians and reptiles could lead to negative human attitudes toward their conservation. Similarly, the welfare of pet rats could be compromised due to their similarity with infamous wild rats, which are often targeted by pest control programs. In addition to the animal itself, pet rat owners might suffer from these negative attitudes as they could be subject to discrimination by society. From interviews of seven pet rat owners, Robin et al. [9] found that they were unable to talk about their pets with people outside the pet rat community since the idea of keeping rats as a companion animal was not widely acceptable. As this rat-human relationship is highly understudied, we set out to focus on pet rats in animal shelters.

Exploring data collected from animal shelters helps us understand the population dynamics of companion animals and guides programs that may improve the welfare of these animals, as well as identify needed support for their owners. Analyzing such data not only clarifies the scope of abandoned or surrendered animals in a community [10], but also helps these animals obtain a better life and increase their adoptability in the future. Many shelter data analysis studies have been conducted for dogs, cats, and rabbits [10–13], but to the best of the authors’ knowledge, no similar studies have been done on pet rodents–rats and mice in particular. In this study, we only focused on rats since they are far more popular than mice and any other animals in the small mammal category [14].

The objective of the study was to describe the population dynamic of rats entering BC SPCA shelters. We wanted to know the reasons why they were admitted and what happened to them after intake. We hypothesized that human-related factors were the most common surrender reason, as is found with other companion animals [4, 13]. To better understand the preferences of their potential adopters, we utilized the data to find predictive factors affecting their likelihood of adoption, as well as their length of stay (LOS) in a shelter. We hypothesized that an old and unhealthy rat that had a dark colouration might be more difficult to rehome because adopters may prefer young and healthy individuals [15, 16], and a brown rat is morphologically similar to a wild rat. A broader goal of this study was to bring more attention to this understudied companion animal within the field of animal welfare and to show some challenges that they might encounter at a shelter.

## Materials and methods

### Subjects and study site

This retrospective cohort study targeted the intake and outcome of rats from 34 BC SPCA animal shelter locations between June 1^st^, 2015 and June 1^st^, 2020. All animal records used had been previously collected by animal care attendants, veterinary technicians, animal cruelty investigators, and other shelter-associated professionals over the 5-year period. The study was approved by the BC SPCA Animal Welfare Research Subcommittee and the University of British Columbia Behavioural Research Ethics Board (H20-01526).

The BC SPCA is the largest non-profit animal welfare organization in the province of British Columbia, encompassing 34 animal shelters, 3 animal hospitals, and 2 spay/neuter clinics. More than 20,000 animals are admitted to the shelters annually and close to 90% of them are either cats or dogs. Other animals, mainly rabbits and rats, only accounted for about 10% [7]. Shelter animals are received from many different sources, including animals transferred from other animal care facilities, owner-surrendered animals, abandoned animals, seized animals from cruelty investigations, and litters from animals who were pregnant at the time of shelter entry.

A portion of animals are admitted to the BC SPCA animal shelter system as “abandoned” or “stray”, which is defined by the *Prevention of Cruelty to Animals Act 1996* (Vic) as an animal that is: “(1) ownerless, (2) found straying, (3) found in a rental unit after the expiry of the tenancy agreement, and (4) not retrieved by a person who has previously agreed to care for the animal within four days”. An owner has four days to find and reclaim their pet if it is found abandoned by the BC SPCA; otherwise, the shelter may adopt out these animals once the holding period has expired. It is important to notice that a wild rat could possibly be misidentified as a stray pet rat; however, as anecdotally suggested by shelter staff, this chance is low since a wild rat can also be easily distinguished from a pet rat through its fearful behavior, morphologically smaller space between ears, and coarse coat.

The cruelty investigation department of the BC SPCA has the authority to enforce animal cruelty laws and takes legal actions in response to animal cruelty cases. Under the *Prevention of Cruelty to Animals Act 1996* (Vic), humane officers may investigate cruelty complaints and seize animals in distress if the search warrant has been approved by judicial justices of the province. These animals will stay “on hold” in a shelter for at least 14 days during which previous owners may appeal for the return of their animals. Once the protective custody period has passed, the shelter may take ownership of the animals and make them available for adoption to the public. In addition to the seized animals, humane officers may receive surrendered animals, which do not require a holding period and can be adopted out instantly if no other concerns are found.

### Data cleansing and analysis

**Fig 1:**
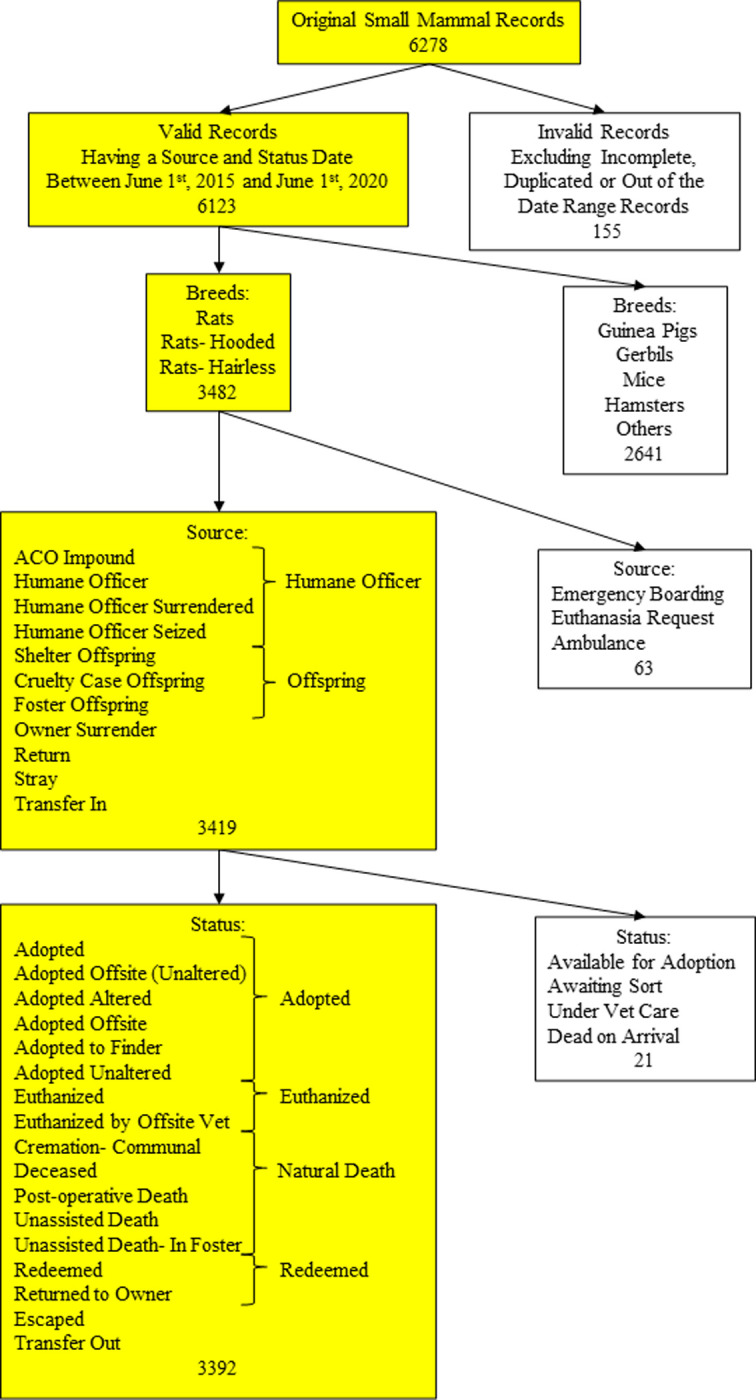
A Flow chart for data collation. Yellow squares indicate the direction of data cleansing.

A data subset of 6,278 small mammal admissions to 36 BC SPCA animal shelters between June 1^st^, 2015 and June 1^st^, 2020 was sourced from ShelterBuddy [17], a database that is shared by all the BC SPCA shelters. Small mammals other than the species of “rats”, “rats-hooded”, and “rats-hairless” were excluded (Fig 1).

Rats that came to the shelter for emergency boarding, owner-requested euthanasia, and urgent medical conditions that resulted in an immediate euthanasia were removed as these occurred infrequently and did not result in animals entering the shelter facility for the purposes of rehoming (Fig 1). Rats with an ongoing status, including individuals that were available for adoption, under veterinary check, or waiting for an adopting decision, were also removed from the dataset because they did not have a final outcome date to determine the length of stay (LOS). Additionally, the source and outcome of the rats were regrouped for the purpose of simplification (Fig 1).

The age of an animal is often acquired from consulting with the surrender owner or estimation by a veterinary technician or animal care attendant. However, according to anecdotal reports by shelter staff, aging a rat is challenging and may be inaccurate as the determination is based on subjective measures of the size, coat condition, teeth wear, and excitability. Therefore, the age of rats was divided into three categories. Since a rat is socially mature when it reaches 6 months of age and its lifespan in the laboratory is about 2–3.5 years [18], we defined rat pups as ≤ 6 months old (i.e., age of sexual maturity [18]), adults between 6 months and 2 years old, and seniors >2 years old (lifespan in the laboratory is about 2–3.5 years [18]).

The reasons for relinquishment of owner-surrendered, humane officer-surrendered, and returned rats were divided into 7 categories with their description listed in Table 1. Only “animal behaviour” was assigned to the “animal-related factors” while the remaining 6 categories were assigned to the “human-related factors” of surrender.

**Table 1:** The reason for relinquishment of rats and the description.

*Surrender Reason*	*Description*
“Abandoned”	Found abandoned, abandoned by a friend, relative, or tenant during a move
“Animal Behaviour”	Bit a person or another animal, under-socialized, destructive behaviours
“Expense Issues”	Unable to afford veterinary costs or general expenses
“Housing Issues”	Unable to find or afford pet-friendly housing, landlord did not allow, moving to a new house, etc.
“Unwanted Litters”	Pregnant animal, unplanned litters
“Owner-related Issues”	Person has allergies to pet, too much responsibility or no longer wanted, problems with other pets or having too many pets, life change including illness/injury/new baby/divorce/travel/school/work, etc.
“Other Issues”	Military transfer, rescue, treatment centre, etc.

The LOS was determined by the difference between the intake and outcome date. The LOS included the days when animals were sent to a veterinary clinic, in foster, or during a transfer because these temporary statuses were difficult to determine and account for due to lack of reporting. Since the intake date was set as Day 0, the LOS for a rat that had the intake and outcome on the same calendar date was calculated as zero days. When calculating the LOS, the holding periods during which the shelter has no authority to adopt, euthanize, or otherwise dispose of an animal under the *Prevention of Cruelty to Animals Act 1996* (Vic) were excluded. Therefore, we excluded 4 days for stray rats and 14 days for humane officer-seized rats if they were adopted eventually. It is important to acknowledge that the holding period for stray rats could vary depending on the local government bylaws. For newborn rat pups, we did not calculate their LOS until they reached 28 days old because rats younger than that were not adoptable.

RStudio version 1.2.5042 was used to calculate descriptive data and conduct further analysis in this study. The descriptive data such as the physical characteristic of rats, source and outcome, reason for relinquishment, and reason for euthanasia were presented by the number of individuals and their percentage. The LOS of adopted and euthanized rats were shown respectively. Only those that were adopted and had complete records of source, sex, colour, Asilomar Accords category, and age were further analyzed by a multiple linear regression to predict their LOS. The adjusted R square, coefficient, standard error, the 95% confidence interval, and p value were reported. Subsequently, Tukey’s HSD tests with Bonferroni correction were conducted as a post-hoc comparison for each statistically significant variable. The results, along with the mean, median, and distributions of the data, are demonstrated on rain cloud plots. A *p-value* of less than .05 was considered as statistically significant.

## Results

### Admission

A total of 3,392 pet rats (including 100 duplicated records due to post-adoptive returns and secondary adoptions) were admitted to the BC SPCA animal shelter between June 2015 and June 2020. Upon arrival to any shelter branch, animals undergo a thorough health check within 24 hours by staff to record their physical characteristics, health conditions, and presence of any contagious diseases and parasites. The number and percentage of rats by the source of intake, physical characteristics, and subsequent outcomes are summarized in Table 2. Approximately 46.5% of the incoming rats had been surrendered by their owners, 19.4% were brought in by humane officers, 19.1% were pups from an owner’s rats, 12.0% came in as a stray, and 2.9% were returned. Of the 658 rats from humane officer intakes, 132 were seized and 526 were surrendered by their owners. Although not included in this table, we note that a majority of the seized rats (80.5%) were identified as involved in cruelty cases that had a single intake of more than 15 rats.

**Table 2:** Breakdown of admission data and physical characteristics of rats upon intake (N = 3,392).

*Category*	*n*	*% Admissions*
Source of Admissions		
Humane Officer	658	19.4
Offspring	648	19.1
Owner Surrender	1577	46.5
Returned to Shelter	98	2.9
Stray	407	12.0
Transfer In	4	0.1
Age^e^		
Pup	1622	47.8
Adult	977	28.8
Senior	140	4.1
Unknown	653	19.3
Sex^a^		
Male	1256	37.0
Female	1251	36.9
Unknown	885	26.1
Sexual Status^a^		
Desexed	52	1.5
Intact	3014	88.8
Unknown	326	9.6
Colour and Appearance^a^		
Albino	489	14.4
Beige	315	9.3
Black	227	6.7
Brown	578	17.0
Grey	443	13.1
Hairless	143	4.2
White	1197	35.3
Outcome^a^		
Adopted	2022	59.6
Euthanized	1089	32.1
Escaped	12	0.3
Natural Death	33	1.0
Returned to Owner	26	0.8
Transfer Out	210	6.2

^a^ includes duplicated individuals that were returned and adopted repeatedly.

Most of the admitted rats were reported to be less than 6 months old (47.8%), followed by 28.8% between 6 months and 2 years old, and 4.1% were reported to be over 2 years old. There was not much difference in rat admission between sexes (male: 37.0%; female: 36.9%). Rats with unknown sex were mostly litters born onsite that were subsequently euthanized right after birth (Unknown: 26.1%). Results from the desex status show that only a few rats at intake were sterilized (1.5%), and a large proportion remained sexually intact (88.9%). Rats came in a variety of different colours, but white rats were the vast majority (35.3%). Other common colours included brown (17.0%), albino (14.4%), and grey (13.1%).

Owners who relinquish animals to the BC SPCA are required to provide a reason for surrender, and a total of 1,926 responses were gathered throughout the 5-year period (Table 3). It is important to note that the total number of responses is greater than the number of owner-surrendered rats because this number also included responses from owners who surrendered their rats to humane officers. Overall, human-related factors (98.6%) were responsible for nearly all of the relinquishment of rats, while animal-related factors, such as unwanted animal behaviour, only accounted for 1.4% of relinquishment. Nearly two-thirds of surrender reasons were due to owner-related issues (61.9%) such as a life change, travel, owner’s health problems, etc. The second most common reason was housing-related (17.6%) as many surrendering owners stated that they could not afford or find pet-friendly housing while moving. Other less common reasons included financial problems (6.0%) and unwanted litters (8.1%).

**Table 3:** Reasons given for relinquishment (N = 1,926).

*Reason*	*n*	*% of Relinquishment*
Abandoned	59	3.1
Animal Behaviour	28	1.4
Expense Issues	115	6.0
Housing Issues	339	17.6
Other Issues	37	1.9
Owner-related Issues	1192	61.9
Unwanted Litters	156	8.1

Approximately half of relinquishing owners (44.7%) reported the original source of their rats (861 responses throughout the 5 years; Table 4). The two most common origins were commercial pet stores or breeders (30.2%) and unplanned litters (30.9%). Other origins included 14.9% that were reported by the owner to have been stray and/or abandoned and 10.6% that originated from animal shelters and rescue organizations other than the BC SPCA.

**Table 4:** Sources of rats reported by relinquishing owners (N = 861).

*Source*	*n*	*% of Total*
Animal Shelter or Rescue Organization	91	10.6
Breeder or Pet Store	260	30.2
Free from the Public	70	8.1
Planned Litter	26	3.0
Stray or Abandoned	128	14.9
Unplanned Litter	266	30.9
Other	20	2.3

### Outcome

The outcomes of the rats are reported in Table 2. Most rats were adopted (59.6%), while about a third were euthanized (32.1%). A small proportion (6.2%) was transferred to other animal care facilities.

The BC SPCA utilizes the Asilomar Accords guidelines for categorizing animal health and behavioural conditions at intake, and while Asilomar category does not necessarily determine animal outcome, there is an association between them [19]. All of the BC SPCA shelters began assigning Asilomar Accords categories to animals as of February 2016. Therefore, some rats entering the shelter before this date were not given a category. The outcomes of 2,487 rats that had been assigned a category at intake are listed in Table 5. Nearly all (94.9%) rats that were assigned a “Healthy” (H) status were adopted and none were euthanized. Both the “Treatable-Rehabilitatable” (TR) and “Treatable-Manageable” (TM) rats had a similar adoption rate (TR: 75.6%; TM: 72.7%). The euthanasia rate of the TR rats (16.5%) was somewhat lower than the TM rats (23.8%), although the result from a Chi-square test did not show that this difference was statistically significant (p = .06). In direct contrast to the H rats, 95.1% of the “Unhealthy and Untreatable” (UU) rats were euthanized.

**Table 5:** Outcomes by Asilomar accord category (N = 2,487).

			*Outcome*			
	*Adopted*		*Euthanized*		*Other* ^a^	
*Asilomar Accords Category*	*n*	*%*	*N*	*%*	*N*	*%*
Healthy (H)	971	94.9	0	0.00	52	5.1
Treatable-Rehabilitatable (TR)	642	75.6	140	16.5	68	7.9
Treatable-Manageable (TM)	104	72.7	34	23.8	5	3.5
Unhealthy and Untreatable (UU)	5	1.1	451	95.1	19	3.8

^a^“Other” included rats that were transferred to animal care facilities outside of the BC SPCA, died naturally, or escaped.

The three main reasons for euthanasia are listed in Table 6. Nearly half of the euthanasia decisions were made due to medical issues including chronic disease conditions (3.3%), upper respiratory disease (4.4%), critical distress (7.6%), and other conditions deemed untreatable in the shelter environment (31.5%). The second most common reason was the lack of space in the shelter (27.1%). Rat pups born onsite were most likely to be euthanized for this reason. Only 20.8% of euthanized rats were euthanized due to behavioural issues such as not being social to people or animals (17.2%) and being aggressive to humans (3.6%).

**Table 6:** Reasons given for euthanasia (N = 1,086).

*Reason*	*n*	*% of Euthanasia*
Medical Issues		
a. Chronic Disease Condition	38	3.3
b. Critical Distress	87	7.6
c. Upper Respiratory Disease	50	4.4
d. Other Untreatable Condition	359	31.5
Total	534	46.8
Behavioural Issues		
a. Aggressive to Humans	41	3.6
b. Not Social to People or Animals	196	17.2
Total	237	20.8
Other Issues		
a. Lack of Space	309	27.1
b. Other	61	5.3
Total	319	32.4

### Length of stay

**Fig 2:**
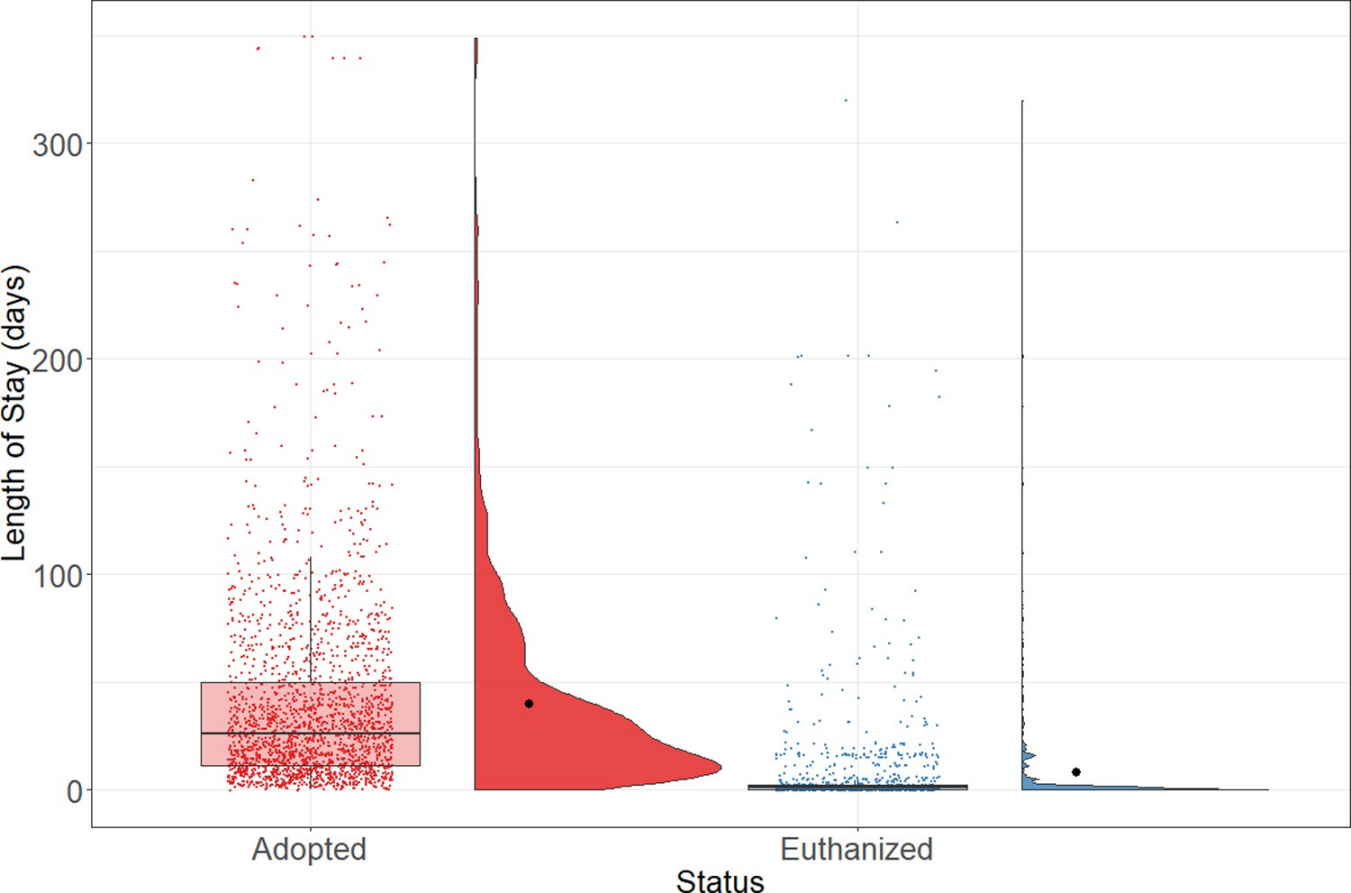
LOS by outcome. Boxplots represent the median, the 25 and 75 percentiles, with whiskers representing the 95 percentiles. Individual data is shown by dots on the boxplot. Additionally, the rain cloud plots show the distribution and the means in each category.

The length of stay (LOS) for animals in a shelter is an important index to describe both the shelter efficiency and the preference of potential adopters. The overall LOS of adopted (median = 26 days; n = 2,022) and euthanized (median = 1 day; n = 1,089) rats are presented in Fig 2.

Factors that affected the LOS of adopted rats were determined by a multiple linear regression using the generalized linear model in the R package. Confounding variables such as circumstance, intake region, and intake jurisdiction were identified through a casual diagram and were removed from the analysis. The final model included source of intake, sex, age, colour, and Asilomar Accords category as predictors. Rats that were not adopted or did not have complete records of the final variables were removed from the analysis (n = 1,479).

Continuous variables, such as age and LOS, were first examined on density plots, and both were visually found to be positively skewed. After performing data transformation, the log-LOS reached normality, but the age variable did not. Prior to conducting the multiple linear regression, the normality of the residuals had been verified by a residual plot from a linear regression that included age as a predictor and log-LOS as an outcome variable. The predefined age groups (pup, adult, and senior) and log-LOS were used in the subsequent Tukey’s HSD test.

Overall, the model showed that the source of intake, colour, age, and Asilomar Accords category, but not the sex of rats predicted their time to adoption (Table 7; F[12, 1466] = 9.565, p < .001, adjusted R^2^ = .06).

**Table 7:** Multiple linear regression model predicting log LOS by characteristics of rats (N = 1,479).

*Characteristic*	*Coefficient*		*SE*	*95% CI*	*F*	*df*	*p-value*
Source of Intake					13.8	3	< 0.001
Humane Officer	Referencexs						
Offspring	0.018		0.053	-0.086 to 0.122			0.73
Owner Surrender	-0.140		0.038	-0.214 to -0.065			< 0.001
Stray	-0.248		0.047	-0.340 to -0.156			< 0.001
Sex					1.4	1	0.23
Male	Reference						
Female	0.037		0.023	-0.008 to 0.082			0.14
Age	2.4e-4		6.6e-5	1.1e-4 to 3.7e-4		1	< 0.001
Appearance					7.2	5	< 0.001
White	Reference						
Beige	0.029		0.039	-0.047 to 0.105			0.44
Black	-0.026		0.044	-0.112 to 0.060			0.56
Brown	-0.019		0.030	-0.078 to 0.040			0.53
Grey	-0.034		0.036	-0.104 to 0.036			0.34
Albino	0.253		0.045	0.165 to 0.341			< 0.001
Asilomar Accords Category					12.4	2	< 0.001
H	Reference						
TM	0.167		0.053	0.063 to o.271			< 0.001
TR	0.097		0.024	0.050 to 0.144			< 0.001

Variables are listed under the characteristic. The coefficient, standard error (SE), the 95% confidence interval (CI), F-value (F), and degree of freedom (df) for each variable along with the p-value are presented.

**Fig 3:**
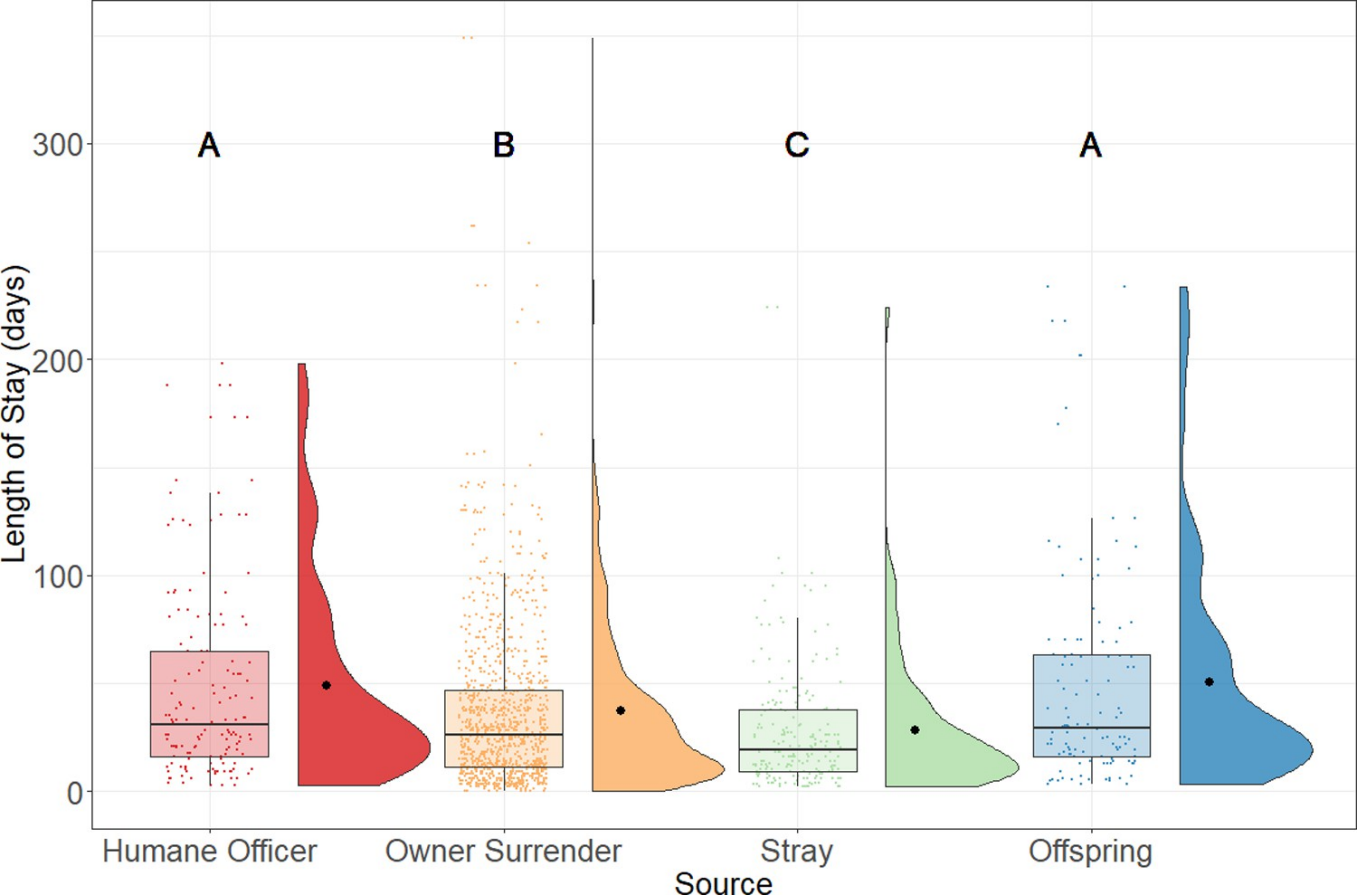
LOS of adopted rats by source of admission. Boxplots represent the median, the 25 and 75 percentiles, with whiskers representing the 95 percentiles. Individual data is shown by dots on the boxplot. Additionally, the rain cloud plots show the distribution and the means in each category.

The rats from cruelty seizures or humane officer surrenders were assigned a “humane officer” source, and this category was used as a reference because it represented the largest proportion of the long-stay rats. Results from the regression model suggested that the LOS of owner-surrendered rats and stray rats were predicted to be 27% and 43% shorter than the LOS of humane officer-surrendered rats. In Fig 3, the humane officer-surrendered rats (median = 31; n = 153) had nearly the same LOS as the offspring rats (median = 29; n = 121; p = .98), but they both had statistically significantly longer LOS than rats that were relinquished by owners (median = 26; n = 1,004; p < .001). Rats that came in as a stray (median = 19; n = 201) had the shortest LOS compared to the other sources.

**Fig 4:**
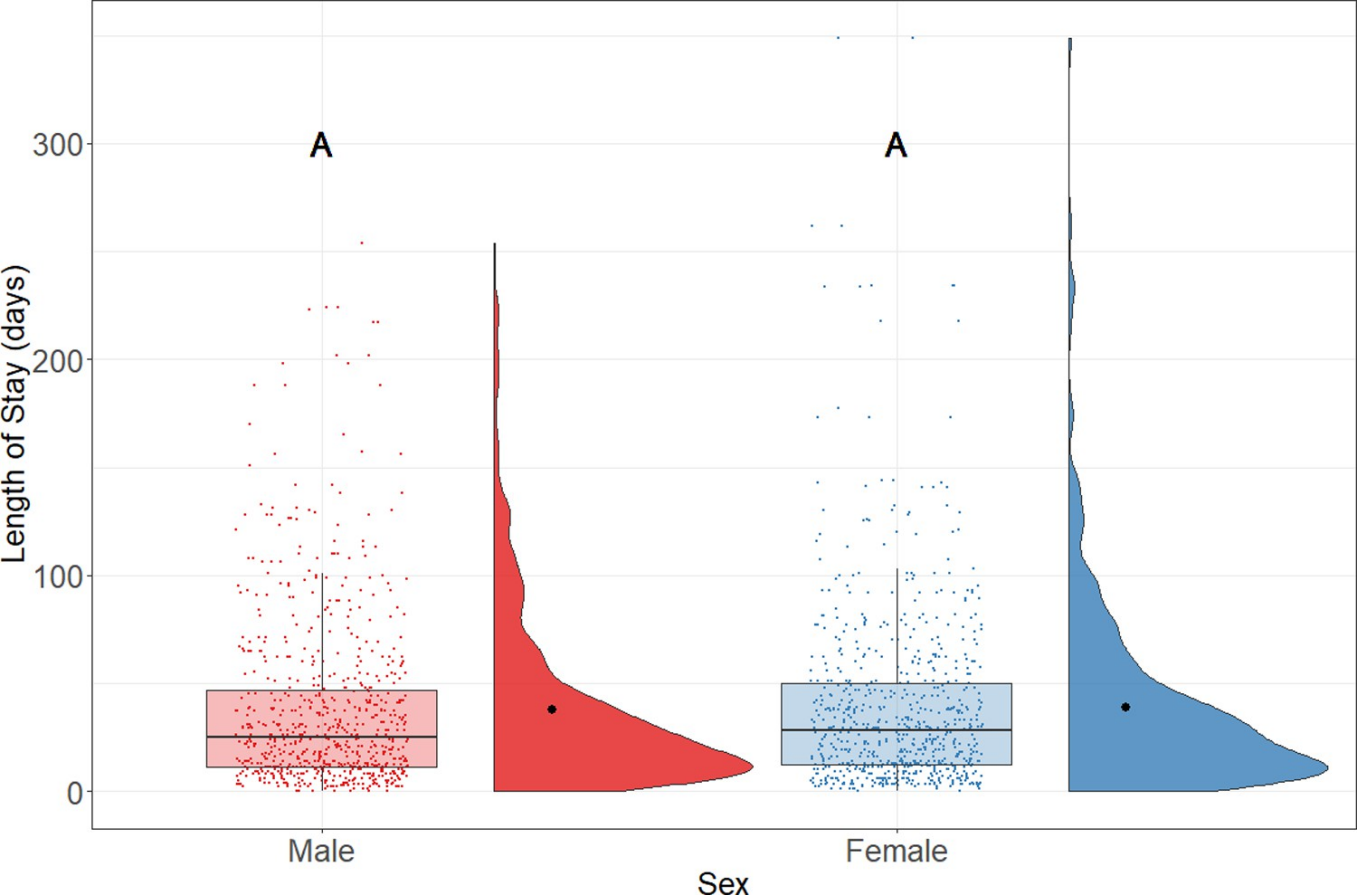
LOS of adopted rats by sex. Boxplots represent the median, the 25 and 75 percentiles, with whiskers representing the 95 percentiles. Individual data is shown by dots on the boxplot. Additionally, the rain cloud plots show the distribution and the means in each category.

The reference group for sex (male) was selected randomly. No statistically significant difference was found between sex and LOS. Results from the regression model suggest that the difference in sex did not predict the LOS of rats. Similarly, as Fig 4 shows, there was no statistically significant difference (p = .24) in LOS between males (median = 25 days; n = 745) and females (median = 28 days; n = 734).

**Fig 5:**
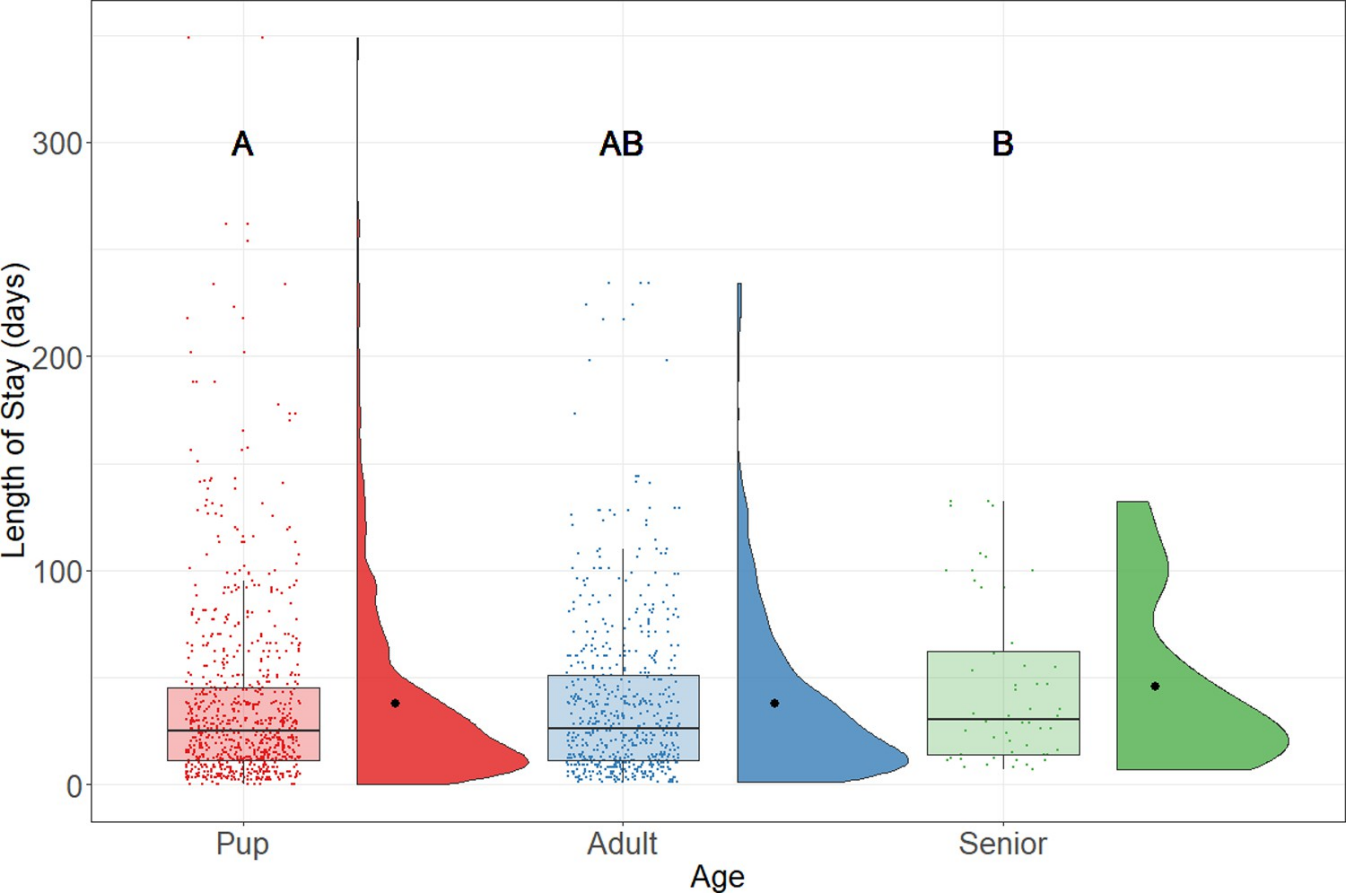
LOS of adopted rats by age. Boxplots represent the median, the 25 and 75 percentiles, with whiskers representing the 95 percentiles. Individual data is shown by dots on the boxplot. Additionally, the rain cloud plots show the distribution and the means in each category.

The age of rats could be used to predict their LOS in the shelter. Results from the regression model suggest that the LOS increased by 0.05% for every day increase in age; this approximates a 20% increase in LOS for every year increase in age. However, the LOS between different age groups was not obvious; as Fig 5 shown, there was no statistically significantly difference (p = .66) between the LOS of the pup (median = 25 days; n = 815) and adult (median = 26; n = 612). The senior rat had the longest LOS (median = 30 days, n = 52) and was statistically significantly different (p = .004) from the LOS of the pups, but this age group also had a much smaller sample size than the others.

**Fig 6:**
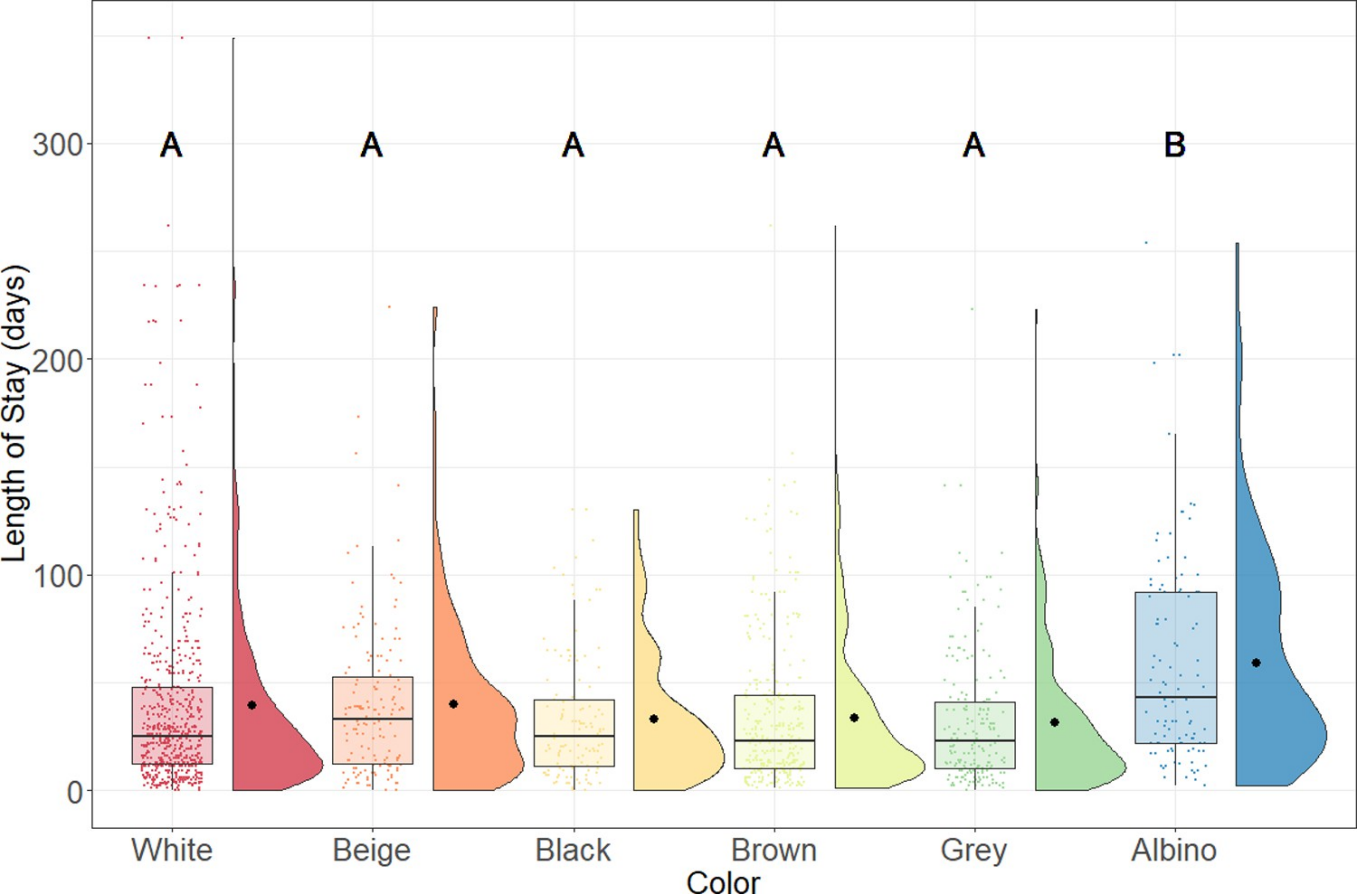
LOS of adopted rats by colour. Boxplots represent the median, the 25 and 75 percentiles, with whiskers representing the 95 percentiles. Individual data is shown by dots on the boxplot. Additionally, the rain cloud plots show the distribution and the means in each category.

The white appearance was selected as a reference group because it represented the majority of the incoming rats. Results from the regression model suggest that the LOS of albino rats was predicted to be 79% longer than for white rats. Similarly, the LOS of albino rats (median = 43 days; n = 107) was statistically significantly longer than the white (median = 25; n = 589; p < .001), beige (median = 33; n = 158, p = .005), black (median = 25; n = 120, p < .001), brown (median = 23; n = 312, p < .001), and grey (median = 23; n = 193; p < .001) rat (Fig 6).

**Fig 7:**
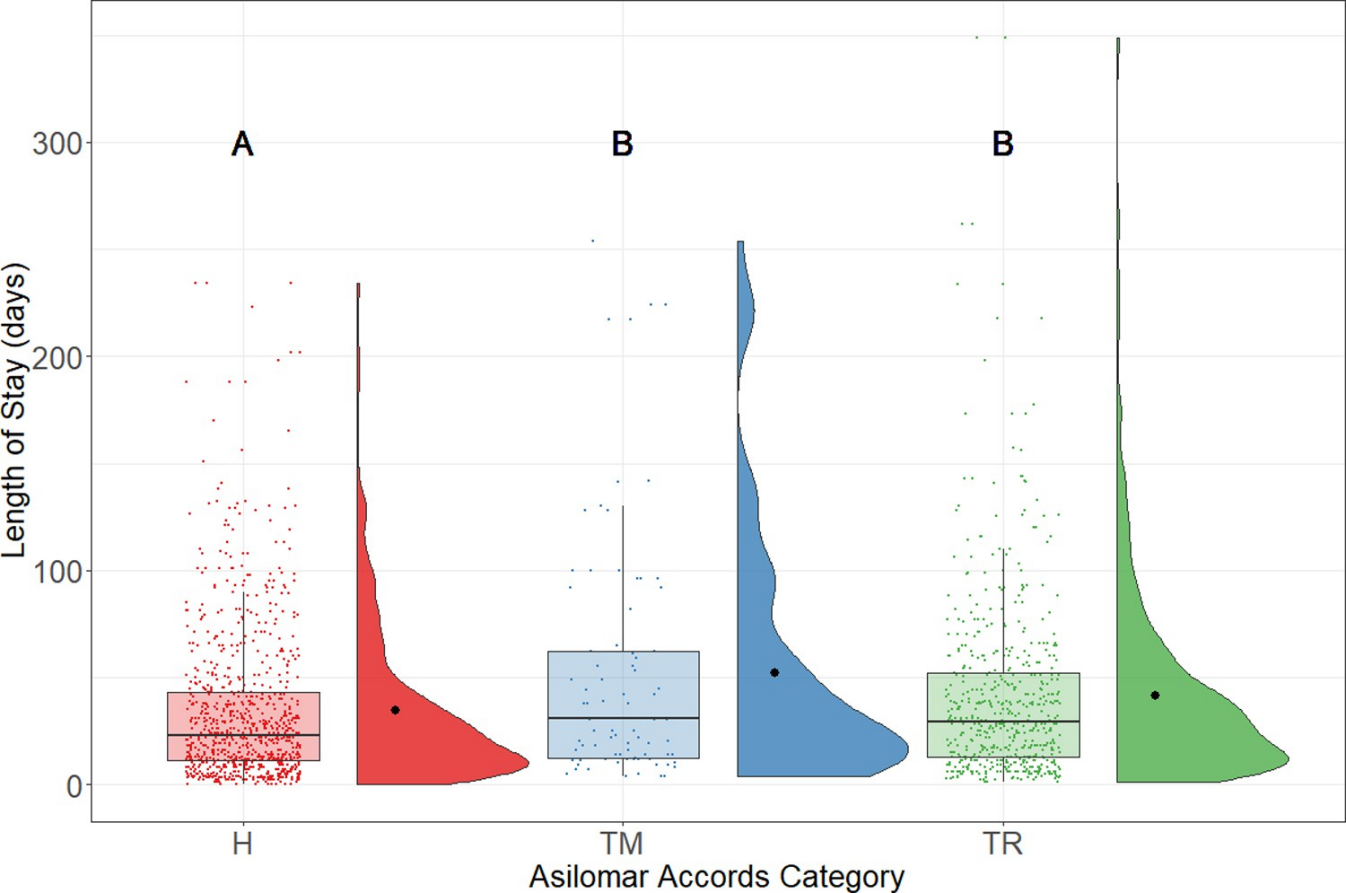
LOS of adopted rats by Asilomar accords category. Boxplots represent the median, the 25 and 75 percentiles, with whiskers representing the 95 percentiles. Individual data is shown by dots on the boxplot. Additionally, the rain cloud plots show the distribution and the means in each category.

The behaviour and health conditions of rats, as indicated by placement into various Asilomar Accords categories, affected their LOS in the shelter. The healthy category (H) was selected as a reference group because it represented the majority of the incoming rats. Results from the regression model suggest that the LOS of rats with a TM or TR status were predicted to be 50% or 26% shorter than the H status. Similarly, as Fig 7 shows, the LOS of healthy rats was statistically significantly shorter than for TM (median = 30; n = 81; p = .003) and TR rats (median = 29; n = 556; p < .001). There were no UU rats in Fig 7 because most of them were euthanized and thus were excluded from this analysis.

## Discussion

### Admission

With a total of 3,392 admissions over the past 5 years, rats have become the third most frequently received animals by the BC SPCA [7]. Internal data provided by the BC SPCA also showed that the number of animals in “other animal category”, which included rats, mice, hamsters, guinea pigs and etc., has been increasing from 1,807 in 2012 to 2,582 in 2019 [4]. In the United States, the population of gerbil, hamster, and other rodent species, has been rising since 2001 and reached a peak of 3.8 million in 2011 [20]. Such an increase in the small pet mammal population could be related to a gradual increase in human density in cities and a decrease in housing space; one study shows that small-sized dogs were preferred over large-sized dogs in higher density areas [21]. Small mammals have become increasingly popular over the last decade possibly because they do not require too much space. Nevertheless, these findings all suggest that more research is needed to better understand the human-animal bond and the welfare states of these small companion animals.

Most rats were surrendered by their owners (46.5%). In Canada, this number is similar to shelter rabbits (47.8%) at 30 shelters surveyed by the Canadian Federation of Humane Societies [11]. In the United States, Cook and McCobb [10] reported that owner surrender was the primary source of rabbit admissions to the shelter, representing 77.3% of all rabbit intakes. The higher surrender proportion of this study is probably due to the difference in time, human population, and geographical regions.

The second most common source of rat intake was from humane officers (19.4%), which can be further broken down into seizures (3.8%) and voluntary surrenders to the officers in the field (15.6%). The proportion of seized rats approximates to those of seized rabbits from other studies: 3.46% [10] and 3.9% [11]. We did not find any studies that reported the proportion of animals directly surrendered to humane officers or animal control officers likely because these animals were usually included in the owner surrender category or other shelters do not have a cruelty investigation department like the BC SPCA.

Our study identified that 530 (80.5%) rats seized by humane officers were from cases involving a single intake of more than 15 rats. In the case of animal hoarding, owners usually possess more animals than they can adequately take care of and have no self-awareness of their inability to provide sufficient care [22, 23]. Therefore, most hoarding cases were uncovered by cruelty investigations rather than direct animal relinquishments to a shelter and might contain more animals per intake than regular surrender cases [24]. Although we could not confirm the reasons behind each case, this finding suggests that rats could potentially be a victim of animal hoarding in British Columbia. While cats and dogs are still the most frequently reported hoarded species [25–27], small mammals are documented in a few hoarding cases [27], which requires further study. Reporting may also be complicated by the possibility that hoarded rats are exterminated by pest control rather than seized by animal shelters.

Our data showed that only 12.0% of the rat admissions were strays, and this approximates to the percentage of stray rabbits (15.6%) found in US shelters [10], but is significantly smaller than the proportion of stray cats and dogs found in most shelters [21, 28–31]. The proportion of stray animals in shelters varies in different geographical regions [32], and it is partially affected by the type of animal species [10]. Cook and McCobb [10] suggested that rabbits had a lower chance to escape from a house because they were sometimes kept in cages, leading to a higher chance of being surrendered by their owners rather than becoming a stray. It is important to acknowledge that stray animals could also come from owners who intentionally released their animals outside as a form of relinquishment. Similarly, rats are typically housed in indoor enclosures, as recommended by the American Society for the Prevention of Cruelty to Animals and the BC SPCA [33, 34]. Therefore, they could not become a stray as easily as dogs or cats that may have regular access to the outdoors. Their small size and possibly lower survival rate due to various predators in the wild could further reduce the likelihood of them being spotted. An alternative explanation could be that the public does not consider seeing a rat outdoors as a reason to call the BC SPCA, since rats occupy several niches in human society including being a pest species and a wild animal.

The offspring category (consisting of rats born in the shelter or in a foster home) accounted for 19.1% of total admissions, and this proportion is much higher than the offspring category for dogs at the BC SPCA [13]. Such a difference is partially due to the success in promoting spay/neuter for cats and dogs [25] and the fact that most people only owned one individual of that species per household [26]. In Canada, most cats (67%) and dogs (76%) are either spayed or neutered [25] as considerable efforts have been made in both education as well as in providing low-cost spay and neuter services. According to Canadian Standards of Care in Animal Shelters: Supporting ASV Guidelines [35], cats and dogs are required to be sterilized before they are adopted from an animal shelter. However, this standard normally does not apply to rabbits and other small mammals. Although the BC SPCA does include spay/neuter surgery for rabbits in their adoption process, rats are not required to be sterilized and rarely qualify for low-cost services. Our data showed that only 1.53% of the rats were sterilized by the time of admission and most of them were not sterilized prior to adoption.

People may not consider the spay/neuter surgery for rats due to their short lifespan and low market value compared to the other companion animals. Other possible reasons may include the inability to find veterinarians to conduct the surgery as a loss of 3 ml of blood can be life-threatening for a rat given its small size [36], the desire to breed their rats, and/or not knowing the benefits that come with the spay/neuter surgery [10]. Additionally, owners may allow breeding to occur accidentally as rats can become sexually mature at the age of 7 weeks when their genitalia are not easily visible [18] and the current recommendation is to house rats in pairs, at minimum, due to their highly social nature [33]. This may create a challenge for an owner to recognize their rat’s sex and increase the chance of housing male and female rats together. As a result, these combined reasons possibly increase the number of accidentally bred rats.

Likewise, more than half of the surrendered rats originated from either unplanned litters or commercial pet stores/breeders. A survey study from the UK identified that 50% of the owned-rats were sourced from a breeder and an additional 40% were from a pet shop [37]. However, this study also identified that only a few rats were self-bred [37], which is different from the result we found in our data. This discrepancy may be due to the differences in people who responded to a survey about their pet rat compared to those that surrendered them to a shelter. The proportion of the rats from unplanned litters was, additionally, much higher than the proportion found in dogs [13], reflecting their low spay/neuter rate.

The study from the UK revealed that rat owners were more likely to own male than female rats [37]. However, this result was not reflected in our data. There were no differences in proportion admitted nor LOS between the sex of rats admitted to the shelter.

Most of the shelter rats were either a pup (less than 6 months) or an adult (between 6 months to 2 years). Not many senior rats (more than 2 years) were found in our dataset. Given that aging rats accurately is difficult in a shelter, it is possible that the staff underestimated their age to help increase their chance of adoption since older animals are less likely to be adopted [13, 38–40]. Whereas aging rats in laboratories can be done through weighing the eye lens or counting endosteal layers in tibia, these invasive methods are not suitable for an animal shelter [18]. It is theoretically possible to measure the growth of molar teeth similar to how the age of cats and dogs is estimated, but this method can only be applied when rats are growing [18]. More research into practical measures and standardized procedures to assist with rat aging in shelters is required.

We found that most rats were surrendered due to human-related reasons and only 1.45% of rats were surrendered because of unwanted behaviours. This low percentage of animal-related factors contrasts to 8–35% found in dogs [13, 28, 41, 42] and 7% found in cats [43], but is closer to 3.4% found in rabbits [10]. Compared to cats and dogs, rats and rabbits are less likely to present obvious behavioural problems like spraying or biting because they are mostly confined to a cage without continuous contact with their owners [10]. Even if they show aggressive behaviours, we speculate that most owners might tolerate problems since rats and rabbits are not likely to present an immediate danger or cause serious injuries to adults and children. However, some UK rat owners did report disliking the bad odour of rat cages as well as being concerned about their rat’s urine marking when out of the cage [37].

The most common human-related surrender reasons were due to owner-related issues, which is a broad category that included a person’s life change, owner health issues, not wanting the rat any longer, going on vacation, etc. This finding is not surprising given that many studies report a similar result in other companion animal species [10, 13, 28]. Interestingly, we found that housing issues were the second most common reason for the relinquishment of rats, and the proportion (17.6%) is even higher than the number found in dogs at the BC SPCA [13]. This result is consistent with a study from the UK, where housing issues (16.1%) were found to be the second most common surrender reason for rabbits [44]. It is logical to assume that people with dogs are more likely to encounter a relocation problem because dogs are often considered less tolerable in a rental property as they are potentially more destructive and noisier, but to our surprise, owners of rats, which likely spent relatively more time in a confined environment [10], also encountered a relocation problem in British Columbia. To clarify this issue, a future study may survey landlords in the province regarding their pet concerns and rental policies.

### Outcome

Like other companion animal species, the outcomes of rats from the BC SPCA were mostly adoption with only a relatively small proportion euthanized. That being said, the euthanasia rate for rats (32.1%) was much higher compared to dog euthanasia (7.56%) at the BC SPCA [13]. This high euthanasia rate was particularly evident in the offspring category given that rat pups born in-shelter were mostly euthanized soon after birth to avoid over-population and to maintain the outflow capacity of the shelter [19, 45]. One alternative is to spay and abort the pregnant rat as the procedure is more humane and would not cause fetal suffering [46], but this is not feasible in most locations due to limited veterinarian capacity within the province. Other alternatives are to assign rat pups to multiple foster homes if volunteers are available and to transfer them more regularly to a higher capacity shelter, but these do not solve the limited outflow capacity across the entire system. A short-term alternative, perhaps less palatable, is to euthanize the pregnant female rather than waiting for her to give birth. Humane euthanasia of young rat pups is rather challenging due to their small size (rodent neonates are resistant to hypoxia during CO_2_ administration, often requiring a secondary physical euthanasia such as decapitation or cervical dislocation [47]); thus, euthanasia of an adult may lead to a more humane death, less distress for the staff that must euthanize, as well as would result in fewer overall deaths. However, an inability to determine the fetus age of the pup could be a challenge as late pregnancy euthanasia poses further ethical and welfare issues. Euthanasia of animals solely for the reason of pregnancy is also against policies as listed in the Asilomar and Adoptability Guidelines [19].

Results from the Asilomar Accords category show that the classification significantly reflects the outcome of rats, with most of the healthy rats (94.9%) adopted and most of the unhealthy and untreatable rats (95.1%) euthanized. Notably, neonates were automatically assigned an Unhealthy and Untreatable status based on the Asilomar Accords [19]. We found that no healthy rats, as determined by shelter staff, were euthanized, reflecting the BC SPCA’s commitment to not euthanize healthy animals.

### Length of stay

The LOS of animals varies significantly by shelter policies other than the intraspecific characteristics that were measured in this study. Brown et al. [38] suggest that the average LOS of dogs in shelters that only euthanize animals for serious medical and behavioural reasons was considerably longer than the LOS of dogs in shelters that would euthanize animals due to space limitations in addition to health and behaviour reasons. Over time, the improvement in shelter policies and adoption procedures, animal health care, welfare practices, and new marketing strategies also help decrease LOS and create better adoption matches [48–50]. Therefore, it is important to take the time and policy factors into account when comparing the LOS of animals between animal shelters in different studies. Considering that the BC SPCA does not euthanize healthy animals as defined by the Asilomar Accords category, we expect that their animals should experience a longer LOS than other studied shelters that euthanize healthy animals. Other policies like the Capacity for Care and foster programs also impact the LOS of animals [50].

In North America, the LOS of rabbits reported by Cook and McCobb [10] was much longer than the LOS of cats and dogs found in other studies [38, 39]. Likewise, our data showed that rats had a long LOS (median = 26 days) in the current study. The distribution of our data was positively skewed, indicating that many rats stayed in shelters for a long period (range: 0–349 days). We compared the LOS of rats in this study to the LOS of dogs in similar shelters found in literature listed by Brown and colleagues [38] and, similar to our prediction, the result showed that the LOS of the rats is longer than the LOS of the dogs found in these databases [38, 42, 51].

Our data allowed us to construct models to predict LOS based on animal factors. We found that rats born in-shelter (“offspring” category) and rats brought in by humane officers had a longer LOS than the rats from the other intake sources. The LOS of neonate rats is consistent with the result found by Kay et al. [13], who reported that neonate dogs also spent the longest time in the BC SPCA animal shelter system. As the authors suggested, one possible reason is that young animals that were either born onsite or brought to the shelter would spend many days in a foster home until they became old enough for adoption. Although we did not count the first 28 days after a rat was born, it is possible that the rat might stay in a foster home longer than we originally expected; and therefore, they tended to have a longer LOS compared to the others. Rats from the humane officer source had a long LOS likely because they spent more time receiving medical care and behavioural modification if they were subjected to hoarding or neglectful environments. In addition, the LOS of these rats could further be increased due to legal requirements, such as delays in court decisions and appeals by previous owners.

In the current study, we did not find any statistically significant difference in LOS between the sex of the rats. Previous research suggested that male cats and female dogs may be preferred by adopters [39, 40, 50, 52]; however, research has largely found mixed effects of sex on animal adoptions [53].

Our finding shows that the age of rats might have affected their chance of adoption. Rats that were younger than 6 months (but not neonates from the “offspring” category) tended to have shorter stays in the shelter than senior rats, and we found an overall positive correlation between age and LOS from the multiple linear regression. This finding is consistent with the results found in cats and dogs from previous studies that show that younger animals tended to be adopted faster [13, 38–40]. However, it is important to note that the age of rats in the current study might not be accurate due to difficulties in determining age.

It is well known that people make adoption decisions based on the animal’s looks [54–56]. Previous literature indicates that morphological variation like size [15, 38, 40], colour [13, 15, 39], breed [38, 50, 54], and coat patterns [39] all play a potential role in determining an animal’s LOS and chance of adoption. In the current study, coat colour/appearance was the only factor to be examined since rats have neither a complicated breed map nor significant variations in size. Our data showed that albino rats, which are strictly described as pink-eyed white rats [57], had the longest LOS among all the colour morphs. This finding is surprising given that the white rats, which had the same coat colour but with dark eyes [57], had statistically significantly shorter LOS than albino rats. It is possible that the colour of an animal’s eyes might have affected people’s decision in adopting rats or even other animal species, but to the best of the authors’ knowledge, no previous study has described the effect of pink eyes on adoption rate or LOS. One thing to note is that the shelter staff might have accidentally grouped some of the albino rats into the white rat category because both of them have the same white coat colour and we did not find a colour definition list that helped the staff distinguish between the two. Therefore, additional research should be conducted to confirm our findings. Nevertheless, this finding might be useful for breeders and scientists that try to adopt out healthy rats as they can avoid breeding/purchasing albino rats to decrease the overall time to adoption. It could also help animal shelters develop new marketing strategies for adopting out albino rats.

## Conclusions

Our study is the first of its kind to describe the admission and outcome of rats in animal shelters and to explore characteristics that might affect their length of stay. Overall, we found that most shelter rats were sexually intact, and many of them were originated from unwanted litters. These findings suggest that better education about avoiding housing unsterilized male and female rats together should be provided by commercial pet stores and animal shelters to their clients, and more widely available and accessible sterilization options should be developed for small mammals that do not have a high market value. Housing issues were a common reason for the surrender of pet rats, suggesting the need for additional research on pet restrictions and pet-friendly housing in order to reduce surrender and support the human-rat relationship. Given that many rats seized by humane officers were associated with a large-scale animal intake, future studies may consider the reasons and welfare concerns of rats as victims of animal hoarding. We found that albino rats had a longer length of stay; animal shelters could use this information to design new marketing strategies that help reduce their time to adoption. Moreover, age may be an important factor that predicts length of stay, but accurately aging rats may be difficult for staff without additional training.

## Supporting information

S1 File 2015–2020 Small mammals. Rat admission dataset.
(CSV)

S2 File Rscript pet rat. Data analysis.
(R) Click here for additional data file.
